# Continuously Operating Biosensor and Its Integration into a Hermetically Sealed Medical Implant

**DOI:** 10.3390/mi7100183

**Published:** 2016-10-09

**Authors:** Mario Birkholz, Paul Glogener, Franziska Glös, Thomas Basmer, Lorenz Theuer

**Affiliations:** 1 IHP, Im Technologiepark 25, 15236 Frankfurt (Oder), Germany; glogener@fh-aachen.de (P.G.); franziska.gloes@th-wildau.de (F.G.); thomas.basmer@hsae.eu (T.B.); 2 Department of Biotechnology, Technical University Berlin, ACK24, Ackerstr. 76, 13355 Berlin, Germany; Lorenz.Theuer@acreo.se; 3 Acreo Swedish ICT AB, Box 787, SE-60117 Norrköping, Sweden

**Keywords:** biosensor, affinity assay, continuous glucose monitoring, BioMEMS, sterilization, metallic housing, titanium, medical implant

## Abstract

An integration concept for an implantable biosensor for the continuous monitoring of blood sugar levels is presented. The system architecture is based on technical modules used in cardiovascular implants in order to minimize legal certification efforts for its perspective usage in medical applications. The sensor chip operates via the principle of affinity viscometry, which is realized by a fully embedded biomedical microelectromechanical systems (BioMEMS) prepared in 0.25-µm complementary metal–oxide–semiconductor (CMOS)/BiCMOS technology. Communication with a base station is established in the 402–405 MHz band used for medical implant communication services (MICS). The implant shall operate within the interstitial tissue, and the hermetical sealing of the electronic system against interaction with the body fluid is established using titanium housing. Only the sensor chip and the antenna are encapsulated in an epoxy header closely connected to the metallic housing. The study demonstrates that biosensor implants for the sensing of low-molecular-weight metabolites in the interstitial may successfully rely on components already established in cardiovascular implantology.

## 1. Introduction

The quantitative determination of concentration levels of metabolites in the human body is an indispensable tool for medical diagnostics and therapy. Great progress has been achieved in recent years for in vitro diagnostics, where the introduction of new biomarkers, the miniaturization of established sensor principles, and the approach of point-of-care testing [1,2,3,4] has enabled a multiple increase in metabolic data per patient and time. Concomitantly, significant cost reductions per measured data point could be enforced via the usage of multi-parameter analytics, microfluidic platforms, and lab-on-chip systems [5,6,7].

The extraction of data from biosensors implanted into the human body turned out even more challenging [8,9,10], although such in vivo metabolic data are rather significant from a medical point of view. For instance, following the concentration transients *c*(*t*) of certain ions, molecules, hormones, immune response related cytokines, and other species of metabolic relevance will allow for the confirmation of a diagnosis or for the tuning of a therapy with respect to the patient’s physiological conditions. In particular, the emerging field of personalized medicine severely deserves implantable biosensors delivering *c*(*t*) data sets from individual patients [11].

The broad utilization of such system is hindered, for one reason, by the complex interdisciplinary approach that is required for their development. Rather different aspects like the biochemistry of the assay, the semiconductor technology of the sensor chip, the microtechnological integration of the implant, the biocompatibility of the materials used, and other technical and scientific aspects have to be considered and might only be tackled by large interdisciplinary teams. A further obstacle is due to the bio-layer produced in the body on implant surfaces [10], which can cause a drift or a corruption of the measurement signal, often in a time-dependent manner, i.e., varying with time after implantation.

In addition, the development and test of implantable biosensors has to overcome high administrative obstacles, since human body implants are meaningfully subjected to strict regulations via various laws and enactments [10]. This holds in a comparable manner for animal models that are frequently used for preliminary tests of human medical implants [12,13]. One of these requirements relates to the sterilization of the implant that has to be demonstrated prior to any in vivo testing. This latter process may become particularly challenging [14], since it has to be performed at the end of the integration process, when sensitive components have already been integrated into the device.

**Figure 1:**
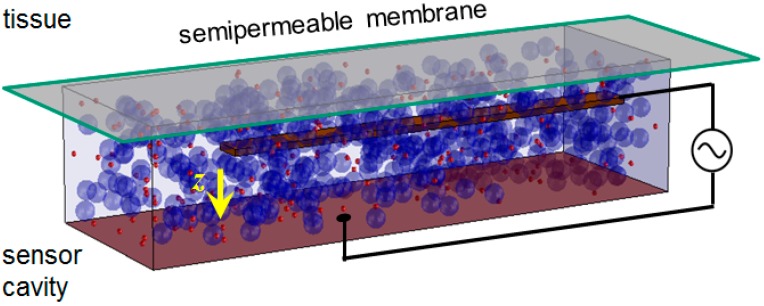
Scheme of affinity sensor microelectromechanical system (MEMS) with reversible binding receptors (small red spheres) and polymers of the analyte (large blue spheres). Analyte molecules (not shown) can pass freely through the semipermeable membrane between tissue and sensor cavity, where they modulate the bonding pattern within the macromolecular network. The state of viscosity is determined by the movement of a bendable beam that is electrically attracted to the ground plate (yellow arrow).

Here, we report on a sensor implant that shall monitor blood sugar levels in humans and transmit the measurement data out of the body. The function of the sensor chips has recently been demonstrated in vitro, where a few % precision in glucose determination could be demonstrated for model solutions [15]. It operates by the principle of affinity viscometry, i.e., it transforms variations of glucose concentration *c_g_* into variations in viscosity η of the biochemical assay (Figure 1). The transformation is performed by virtue of the macromolecules dextran and concanavalin A (ConA), from which the first is a glucose polymer and the second is a plant lectin with a specific binding site for reversible binding of *D*-glucose, i.e., it acts as a receptor molecule. As a lectin, ConA is a carbohydrate binding protein, which has been found to be highly specific for binding to glucosyl and mannosyl residues. The change in viscosity is detected by a microelectromechanical system (MEMS), in which a mechanical beam is deflected quasi-electrostatically, and its deflection velocity is determined within the assay.

**Figure 2:**
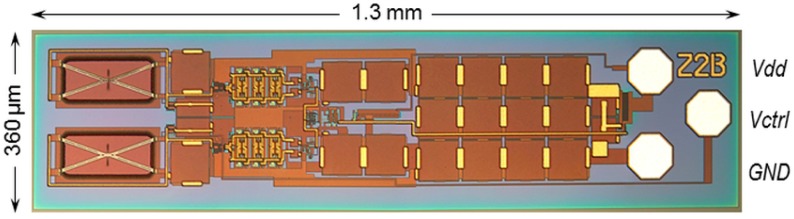
Affinity-viscometric sensor chip with an X-shaped mechanically bendable beam on the left side (bottom: reference; top: measurement) [15].

The space above the MEMS is filled with the assay and is separated from the body tissue by a semipermeable membrane being permeable for low-molecular-weight metabolites, but not for dextran and ConA. A cross-linking network of macromolecules with varying viscosity is formed in the assay, the degree of which depends on *c_g_*. The measurement is not performed in a vessel of the blood circulation, but in the interstitial tissue, where the implant will be situated. The sensor chip is operated by a DC voltage *V_dd_* between 2.5 V and 3.3 V, which is converted to a 3.2-GHz HF voltage in a ring oscillator circuit. The frequency *f*_0_ is chosen to lie between the absorption maxima of proteins and water in protein–water solutions. It is far above any mechanical resonance frequency of the MEMS beam, making it operate in the aperiodic damping regime. There are two MEMS active on the sensor chip that disposes of a deflective and an unbendable beam representing the measuring MEMS and the reference MEMS, respectively [15]. The frequency of the first changes during the measurement, which is followed by a phase-frequency detector (PFD). The time it takes for the beam to deflect to a defined position is taken as a measure for the viscosity of the assay and, therefrom, for the glucose concentration. A chip photo is displayed in Figure 2.

Previous investigations have shown that the exposed surfaces from TiN [16,17], SiO_2_, and SiON remain intact in vitro and would allow sensor chip operation in liquid environments for many months [13,18]. It is shown here how the developed BioMEMS can be integrated into a hermetically sealed implant.

## 2. Integration Concept of the Implant

Because of strict legal regulation of medical implants, all of its technical components are subject to thorough assessment procedures for both clinical trials as well as for a perspective product licensing. Due to this reason, the conception of a biosensor implant made use of technical components—whenever possible—that are already well established in implantology. This was believed to keep administrative permission procedures to a minimum; in fact, various technology modules are commercially available that are routinely integrated into cardio-implants.

This study endeavors to realize a concept. A Ti casing was designed for this purpose by a commercial 3D CAD tool (Solid Works), which had to fulfill various constraints with respect to sensor integration, energy supply, and data transmission. It was not the goal of this study to test the fully integrated device, which remains a more elaborate investigation that will also require the consideration of comprehensive legal procedures.

**Figure 3:**
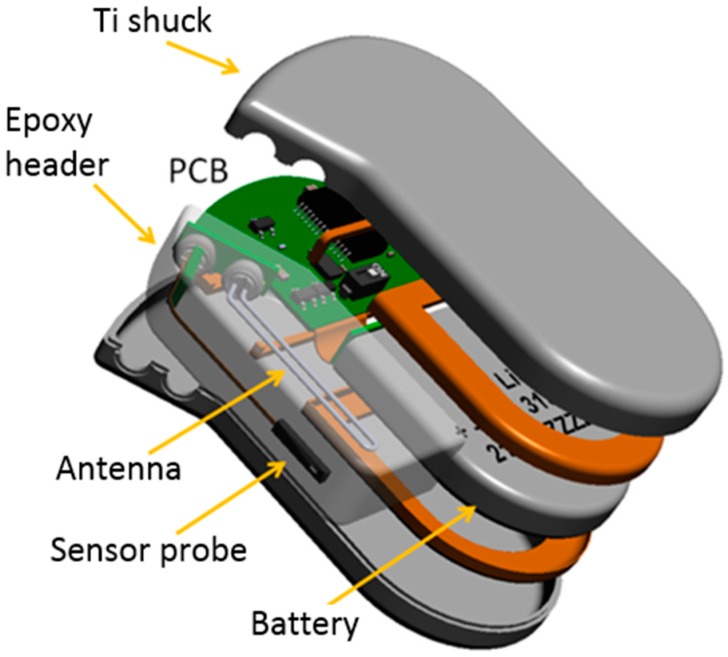
Explosion scheme of biosensor implant in a Ti/epoxy housing.

The basic components of implantable biosensors are the sensor device, the energy supply, data transmission, and the system control [19]. Energy might be delivered by a Li-MnO_2_ battery exhibiting energy densities on the order of 1 Wh·cm^−3^ and which are commonly used in pacemakers and defibrillators. Their performance was found to be in accordance with the intended use cases of the sensor implant, when one *c_g_* measurement shall be performed every 5 min [20]. Figure 3 shows the explosion scheme of the full system, in which the position of one of the prevalent batteries has been indicated. It can be recognized that the battery is the size-determining component of the full system.

Data transmission is preferably performed wirelessly; here, the definement was made to utilize the 402–405-MHz MICS band (medical implant communication service). This ultra-low power band has been commissioned by European and US authorities for the communication between medical implants and a base station [21]. Previous ex situ investigations have demonstrated that data transmission ranges in the MICS band are on the order of 5 m for emissions from body phantom liquids [22]. The selected radio module was the ZL 70321 that has been manufactured by Zarlink until 2011 and subsequently by Microsemi [23].

The control of measurement cycles, data storage, and data transmission is managed by a conventional microcontroller MSP430 (Texas Instruments, Dallas, TX, USA). A three-layer printed circuit board (PCB) with a surface of ca. 5 cm^2^ was designed for the reception of the radio module, microcontroller, and additional passive devices. Its form factor followed from the constraint of being positioned within the housing on the same level, but in opposition to the battery. This was believed to allow for a convenient electrical connection of both.

The most severe challenge that has to be addressed with an electronic system in a biological environment is the hermetical sealing of all components that must not come in contact with the bio-milieu. On the other hand, the sensor has to interact with the tissue via defined active areas.

For a biomedical glucose monitor, it appears reliable to pursue an operational time span of a few months at least, in order to balance costs and efforts for implantation and later explanation. The hermetical sealing of implants for such time spans is only enabled by the utilization of metal housings [8]. For this purpose, titanium has found wide dissemination for the housing of cardio-implants.

The housing to be used here was thus produced from two shucks of titanium grade 5 as usually applied in medical implants. Figure 3 displays an explosion scheme of the designed body implant including the housing as well as the internal components. The electrical connection with the sensor probe and antenna was realized by metal-ceramic feedthroughs. Both the sensor probe and antenna were integrated in an epoxy casting as used in cardio-implants, where it is generally denoted as header.

## 3. Realization

The integration of sensor probes requests a substantial portion of the full system integration. Sensor chips are being prepared by IHP’s proprietary 0.25-µm SGB25V technology [24] on 200-mm CZ-Si wafers and subsequently have to be thinned to 150 µm and chemically etched in order to release the TiN-made MEMS beams from the surrounding isolating dielectric [25]. The last cleaning solution has to be dispelled by liquid CO_2_ in a critical point drying (CPD) step to avoid static friction (stiction) of the beam to the ground plate [26]. In addition, sensor dies have to be separated from the wafer by a laser-assisted cutting process [27], and Au stud bumps have to be placed upon the three bond pads for *V_dd_*, *V_ctrl_*, and ground potential (*GND*) to enable the electrical connection with a flexible PCB via a flip-chip bonding process.

The obtained chips-with-flex modules are glued into a cooling body for dissipating the heat produced during one measurement cycle and which is also fabricated from silicon. A measurement chamber with a free volume of about 1 µL is produced by gluing the semipermeable membrane with a cut-off of 2.2 nm [28] onto the cooling body. The biochemical assay (for precise composition, see [15]) is enclosed within the measurement chamber in subsequent filling and sealing steps.

The integration of the sensor chip into the cooling body and its subsequent filling turned out as the yield-limiting step during sensor build-up. Sensor chips were subjected to stiction whenever air bubbles were formed in the initial filling step. MEMS chips are already known to suffer from reduced yield during their integration into a full system. It seems that this rule has even greater impact for BioMEMS’s that contain a biochemical fluid component in addition to an elastic mechanical beam.

The sensor probes obtained were subjected to test their performance for the determination of glucose levels in vitro before integration. A detailed description of the measurement set-up can be found in [15,28]. The effectively measured quantity of the sensor MEMS is a switching time *t_sw_*, indicating the time it takes for the mechanically bendable beam to deflect to a defined position. The derivation of analyte concentration or glucose concentration *c_g_* is performed via a calibration procedure, from which the coefficients *k_i_* of the characteristic function

**Figure 4:**
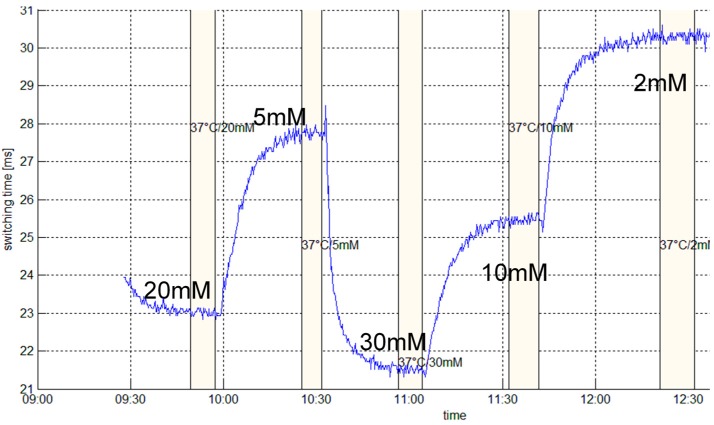
Calibration measurement for determining the switching time *t_sw_* in dependence of glucose concentrations *c_g_*, i.e., 20, 5, 30, 10, and 2 mM of glucose, respectively, in standard electrolyte.

*t_sw_* = *k*_1_ exp(−*c_g_*/*k*_2_) + *k*_3_ (1)

are drawn. Various sensor probes were configured and subjected to glucose sensing tests in vitro in order to demonstrate their reproducibility. Figure 4 displays a typical calibration measurement, from which the numerical values of coefficients *k*_1_ = 11.1 ms, *k*_2_ = 11.4 mM, and *k*_3_ = 20.9 ms were derived. These results compare very well with previous investigations [15,28].

Sterility within the measurement chamber is established by adding cytotoxic agents like HgCl_2_ or NaN_3_ (0.1 wt %), as used for disinfection. Finally, the assay is compacted within the measurement chamber in order to enable a dry handling and storage of sensor probes.

The Ti housing was fabricated by a commercial supplier (Osypka, Rheinfelden, Germany). Integration of components was started by electrically connecting the battery with the system board. The chosen battery (MST Litronik LiS 3150 M) operates with a voltage of nominally 3.2 V and disposes of a capacity of 1200 mAh while exhibiting a volume of 3.68 cm^3^. These performance parameters enable the supply of the sensor and the system board with sufficient electrical energy for a time span of more than six months, when one *c_g_* value is determined every five minutes [20].

**Figure 5:**
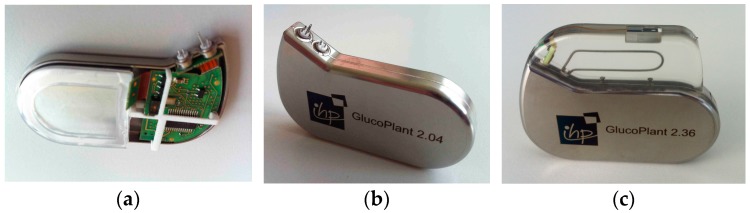
Biosensor implant (**a**) prior to and (**b**) after closing the Ti shucks by laser welding; (**c**) Fully implantable system including header with antenna and sensor probe (59 mm × 45 mm × 8 mm).

Electrically connected battery and board were inserted in the bottom Ti shuck into which the metal-ceramic feedthroughs (MST 4-pol QP/MS & 2-pol BP/MS) were already integrated by laser welding along one half of their full circumference (Figure 5). After connecting the system board with the feedthrough pins for the sensor and antenna, the top shuck was put upon the composite, and the Ti housing was closed in the next welding step. The sensor probe is connected through an extra PCB with the external pins of the 4-pol feedthrough, while the Ti antenna wire is soldered with the pins of the 2-pol. Finally, the probe and antenna are both casted into the epoxy header (Epotek 301, Epoxy Technology Inc., Billerica, MA, USA).

The sterilization of the biosensor implant may be performed neither by steam at 120 °C in an autoclave nor by dry heat between 160 °C and 180 °C, since the enhanced temperature would cause a denaturation of receptor molecules and consequently a de-functionalization of the sensor system. The sensitivity of macromolecules in the assay also prohibits the usage of γ radiation with doses in excess of 20 kGy as often applied for the sterilization of medical implants. Thus, only the usage of UV radiation and ethylene oxide remain as possible sterilization procedures. Our investigations have shown that sensor probes are not affected by UV doses up to 1 J·cm^−2^.

Thus, a combined sterilization process is intended that makes use of the disinfection of the measurement chamber by cytotoxic agents and of the outer surfaces of the implant by UV radiation. Afterwards, the implant is sealed in a sterile package, from which it is released shortly before implantation. An impairment of the patient’s health is excluded, since the implant is bathed in sterile saline prior to implantation, and disinfectant agents in the measurement chamber may be diluted below any desired concentration level.

## 4. Conclusions

We have shown the micro-integration of an implantable biosensor that benefits from a microelectronic sensor chip and operates by the principle of affinity viscometry. It can be concluded that various technical components may be successfully adopted from well-established cardio implants. A particular challenge for affinity biosensor implants is due to the integration of the liquid assay, for which a routine engineering process is required. For the case of an affinity-viscometric sensor presented here, a two-step sterilization procedure has been developed.
